# Duchenne Muscular Dystrophy in Two Half-Brothers Due to Inherited 306 Kb Inverted Insertion of 10p15.1 into Intron 44 of the Dp427m Transcript of the *DMD* Gene

**DOI:** 10.3390/ijms252211922

**Published:** 2024-11-06

**Authors:** Wayne M. Jepsen, Andrew Fazenbaker, Keri Ramsey, Anna Bonfitto, Marcus Naymik, Bryce Turner, Jennifer Sloan, Nishant Tiwari, Saunder M. Bernes, Derek E. Neilson, Meredith Sanchez-Castillo, Matt J. Huentelman, Vinodh Narayanan

**Affiliations:** 1 Center for Rare Childhood Disorders (C4RCD), Translational Genomics Research Institute (TGen), Phoenix, AZ 85004, USA; wjepsen@tgen.org (W.M.J.);; 2 Phoenix Children’s Hospital, Phoenix, AZ 85016, USA

**Keywords:** Duchenne muscular dystrophy, DMD, rare disorders, WGS

## Abstract

Duchenne muscular dystrophy (DMD) is a rare genetic disorder caused by the absence of a fully functional dystrophin protein in myocytes. In skeletal muscle, the lack of dystrophin ultimately results in muscle wasting and the replacement of myocytes with fatty or fibrous tissues. In the heart, cardiomyocytes eventually fail and cause fatal cardiomyopathy. We present a case of a male patient and his younger brother with a maternally inherited inverted insertion of approximately 306 kb of chromosome 10 in the deep intronic region between exons 44 and 45 of the *DMD* gene, leading to Duchenne muscular dystrophy. Chromosomal microarray, comprehensive muscular dystrophy genetic testing, and whole exome sequencing were negative. Targeted transcriptome RNA sequencing at an external lab showed no aberrant splicing. Research whole genome sequencing identified the copy number gain and insertion. Subsequent reanalysis of the RNA sequencing data showed possible aberrant splicing involving *DMD* exons 44–45, and research RNA sequencing revealed a fusion between the *DMD* gene on the minus strand of chromosome X and the *PFKFB3* gene on the plus strand of chromosome 10. We demonstrate that whole genome sequencing can be valuable for identifying intronic events in the *DMD* gene previously undetected or not reported by traditional clinical testing.

## 1. Introduction

Duchenne muscular dystrophy (DMD) is a fatal disorder caused by the absence of a fully functional dystrophin protein in myocytes due to a mutated *DMD* gene, which encodes the dystrophin protein [1]. The normal dystrophin protein features an N-terminal actin-binding domain that anchors the protein to the cytoskeleton of myocytes, as well as a C-terminal dystroglycan-binding domain that anchors the protein to transmembrane proteins [2]. The residues between the termini consist of mainly spectrin-like repeats and hinge regions that lend elasticity to the protein [3]. The full-length dystrophin protein has been shown to help prevent degradation of the cell membrane when myocytes contract, preventing tears from forming, which can cause an exchange of inter- and extracellular material [4].

In skeletal muscle, the lack of functional dystrophin ultimately results in muscle wasting and failure due to these tears in the membrane, which lead to the replacement of myocytes with fatty or fibrous tissues [5]. Calcium ions (Ca^2+^) exist in high concentrations outside of myocytes, and the uncontrolled influx of Ca^2+^ can activate large concentrations of intracellular proteases, which can incorrectly target normal healthy proteins for degradation [6]. Furthermore, creatine phosphokinase (CPK or CK), which is typically found in high concentrations within myocytes and is involved with energy shuttling within the cell, leaks into the extracellular space and can eventually be observed in high concentrations in the blood, a hallmark of the muscle damage caused by DMD [7]. This lack of CPK in the muscle cells is believed to further accelerate the wasting of muscle fibers due to severely inhibited energy maintenance of the cell [8]. In the heart, via the same mechanisms, cardiomyocytes eventually fail and are replaced by necrotic tissue, which severely impairs the pump function of the heart and results in fatal cardiomyopathy, generally in the third decade of life [5].

As an X-linked recessive disorder, DMD predominately affects males (approximately 1 in 5000–6000 male births) and is rarely observed in females (less than 1 in 1,000,000 female births) [1]. The disorder is usually diagnosed between 3 and 6 years of age when difficulty standing or walking becomes apparent and begins to show developmental regression [1]. It is believed that around one-third of DMD cases occur de novo, with the other two-thirds being caused by inheritance from unknowing carrier mothers with no clinical phenotype [9]. A variety of mutation types have been shown to cause the disorder, including deletions, duplications, frameshifts, nonsense, splice-site, missense, and, rarely, translocations [9].

Here, we present two half-brothers who presented with highly suspected cases of DMD. The proband was subjected to several genetic tests at outside laboratories, including a comprehensive muscular dystrophy/neuromuscular panel, targeted transcriptome sequencing of a muscle biopsy, chromosomal microarray (CMA), and whole exome sequencing (WES). His affected half-brother also had a comprehensive muscular dystrophy panel performed. No test results could identify the cause of the muscular dystrophy.

PCR-free whole genome sequencing was performed at the Translational Genomic Research Institute (TGen), part of City of Hope (Phoenix, AZ, USA), which included the mother, proband, half-brother, and their fathers. An approximately 306 kb inverted insertion of 10p15.1 in intron 44 of the *DMD* gene was identified as the causal variant, which was confirmed by the Greenwood Genetic Center (Greenwood, SC, USA) via distal and proximal breakpoint sequencing. The mother was heterozygous for this insertion, and both boys were hemizygous. There was no evidence of this insertion in either of the fathers. This case report highlights the limitations of targeted genetic testing in the absence of a comprehensive approach like WGS when the biological cause is rare, even in disorders as well understood as DMD.

## 2. Case Presentation

### 2.1. Proband and Family History

A 6-year-6-month-old, full-term male patient with a history of left femur fracture presented for lack of ambulation despite having fully healed. He had a prior history of motor delay, urinary incontinence, frequent falls, intermittent leg pain and weakness, and trouble walking up stairs. He did not walk until 2 years of age. There were no neurodevelopmental concerns, and prenatal history was unremarkable.

Given this medical history, the patient underwent initial bloodwork that included CPK, thyroid stimulating hormone (TSH)/thyroxine (T4), and a comprehensive metabolic panel (CMP). The only notable finding was that the patient’s creatine phosphokinase (CPK or CK) test resulted in a significant elevation of 12,581 IU/L, which is 54× to 359× the normal levels (normal range: 35–232 IU/L [7]). Subsequent echocardiogram and electrocardiogram were normal.

To further evaluate the proband, a buccal swab was collected and sent out for next-generation sequencing of 230 neuromuscular genes. This resulted in three variants of uncertain significance, two in *MYL2* and one *TMEM43*, none of which explained the patient’s phenotype. No variants were identified in the *DMD* gene.

Excision of the left vastus medialis oblique was then performed, and tissue was sent to pathology, where it was examined and frozen for further use. The pathology report indicated loss of expression in dystrophin 1 (mid-rod domain) and 2 (C-terminus) with partial loss of expression in dystrophin 3 (N-terminus), consistent with a diagnosis of muscular dystrophy.

Due to the discrepancy between pathology and genetic testing, frozen muscle tissue was sent for targeted transcriptome sequencing. This was performed on an Illumina NovaSeq6000 platform, and sequence data were analyzed using Dragen Bio-IT Platform v3.10.8. The results did not identify any aberrant splicing events. However, subsequent manual review of the sequence data raised concerns about low expression of the canonical DMD transcript (Dp427m/NM_004006). Though the testing platform was limited, it was noted that there was potentially differential expression of a non-canonical dystrophin transcript, specifically the Dp140 (NM_004013) isoform.

Family history was significant for the proband’s father and maternal half-brother. By report, the proband’s father experienced two separate left femur fractures in his childhood and carried a diagnosis of fibrous dysplasia. The proband’s maternal half-brother presented after the proband at 2 months of age with elevated serum CPK of 44,130 IU/L (190× to 1260× the normal range) and was later diagnosed with generalized weakness, at least one bone fracture, and a language delay—Duchenne muscular dystrophy was strongly suspected. A muscular dystrophy gene panel identified two benign pseudodeficiency alleles in the *GAA* gene. His prenatal history was unremarkable, and he has not yet shown any symptoms of muscular dystrophy.

### 2.2. Insertion Detection

**Figure 1:**
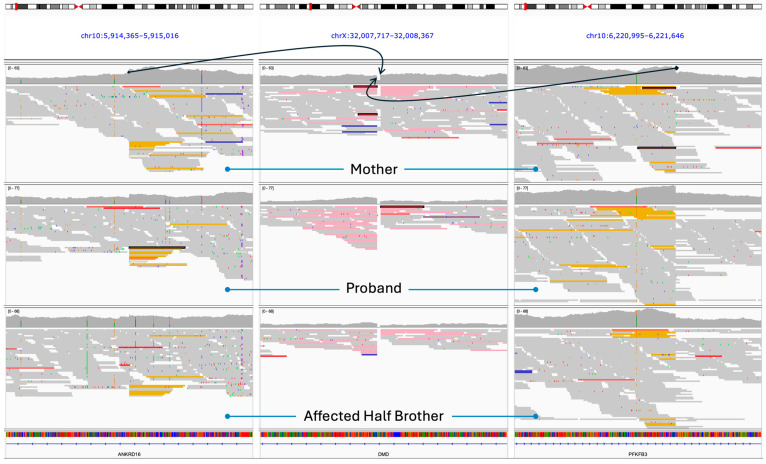
IGV screenshot of the whole genome sequencing data from the mother, proband, and affected half-brother. The arrows indicate where the duplicated region of chromosome 10 (**left** and **right** panels) read through the breakpoints on chromosome X. The yellow reads on chromosome 10 correspond to the pink read mates on chromosome X. Note that the duplicated region starting at 10:5,914,692 reads through X:32,008,035 and the duplicated region ending at 10:6,221,421 reads through X:32,008,026 indicating an inverted insertion.

Utilizing the whole genome sequencing (WGS) data, manual analysis of the *DMD* gene revealed an 8 bp deletion in intron 44. Further investigation revealed that the reads that flanked the deletion had read mates that mapped to the p-arm of chromosome 10 (10p15.1) with distinct duplication points 306,730 bp apart. Reads upstream of the first chromosome X breakpoint, X:32,008,026 (GRCh37/hg19), had read mates that mapped to the breakpoint at 10:6,221,421 (GRCh37/hg19), while reads downstream of the second chromosome X breakpoint, X:32,008,035 (GRCh37/hg19), had read mates that mapped to the breakpoint at 10:5,914,692 (GRCh37/hg19). No other translocation breakpoints were found upstream, within, or downstream of the chromosome 10 breakpoints. This strongly implied that this portion of chromosome 10 is duplicated within the X chromosome and inserted in an inverted manner (see Figure 1). The insertion was confirmed by an external laboratory (Greenwood Genetics Center, Greenwood, SC, USA) by sequencing the proximal and distal breakpoints.

### 2.3. CNV Analysis

**Figure 2:**
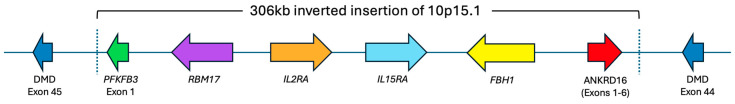
The diagram above portrays the inverted insertion within the *DMD* gene (not to scale). The arrow directions indicate the 5′ to 3′ direction of the labelled gene or exon, with arrows pointing left indicating a gene on the minus strand, and arrows pointing right indicating a gene on the plus strand of the mutant X chromosome. Each gene is colored uniquely to improve readability. The vertical dashed lines indicate the breakpoints on the X chromosome.

To confirm the duplication implied by the manual analysis, CNVkit v0.9.10 was used to informatically identify copy number variants. It identified a copy number duplication in a region just inside of the identified breakpoints. This confirmed that both boys and the mother had three copies of this region of 10p15.1 in their genomes, though the specific coordinates of the copy number variant do not perfectly match those implied by the structural variant caller due to CNVkit’s analytical methods. While the original CMA performed on the proband’s blood at GeneDx (Gaithersburg, MD, USA) was returned with a result of “Negative” due to no pathogenic copy number variants being identified, a second review of the raw data confirmed that the CNV identified at TGen was present in the CMA but did not meet their criteria for reporting as pathogenic. The region of duplication includes the following six protein-coding genes: partial *ANKRD16* (minus strand), *FBH1* (plus strand), *IL15RA* (minus strand), *IL2RA* (minus strand), *RBM17* (plus strand), and partial *PRKFB3* (plus strand). Because of the inverted insertion, all strands are reversed in the final chromosome X assembly (see Figure 2).

### 2.4. RNA Sequencing

**Figure 3:**
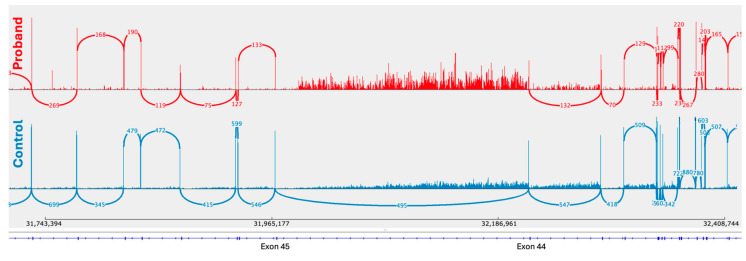
Sashimi plots displaying the splicing of the DMD transcript in the proband’s muscle tissue (red) compared with the control muscle tissue (blue). Note that there are no reads spanning and reading through exons 44 and 45 in the proband’s data.

RNA sequencing of the proband’s muscle biopsy was performed at TGen to confirm the disruption of the dystrophin transcript. Sashimi plots were used to compare the proband’s results to a control muscle tissue RNA sample (see Figure 3). The data implies partial intron 44 retention of the *DMD* gene as it approaches the breakpoint with no reads spanning exon 44 and 45.

**Figure 4:**
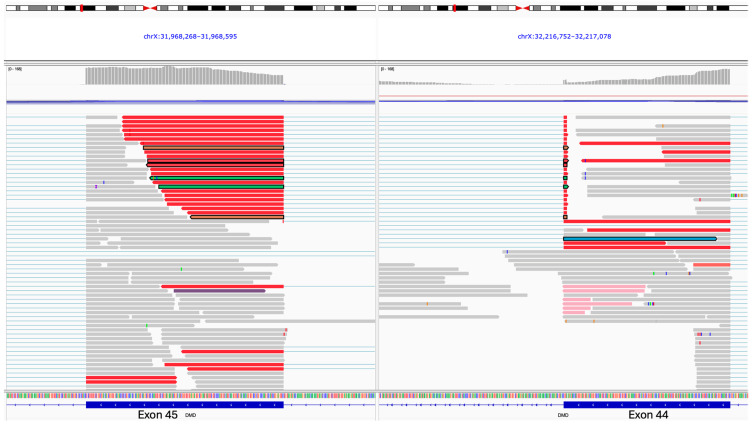
IGV screenshot of the reads appearing to span exons 44 and 45 of the *DMD* gene are displayed in split screen mode. Note that the reads that appear to span the junction map just three bases to exon 44, and there are zero reads that span the junction and read further through exon 44, indicating that these reads are false calls, which is why they were removed from the sashimi plots.

It is worth noting that 25 reads were observed spanning the splice junction in question (exon 44 to 45); however, upon manual inspection of the data, it was determined that these were false alignments of just 3 bp mapping to the splice donor site of exon 44 with no reads through of the exon (see Figure 4). Therefore, the sashimi plot for the proband was filtered to remove any junctions with 25 or fewer spanning reads, which revealed the complete disruption of the *DMD* gene.

The RNA fusion data identified a single fusion transcript, *PFKFB3*::*DMD*, with breakpoint at chrX:31968514 (GRCh38/hg38), which is the start of exon 45 of the *DMD* gene (Dp427m/NM_004006.3) and at chr10:6145013 (GRCh38/hg38), which is the end of exon 1 of the *PFKFB3* gene (NM_001145443.3). These data are consistent with the location of the breakpoints and the orientation of the insertion identified in the WGS data and potentially explain the sashimi plot downstream of exon 45: the native splice sites of the *DMD* gene are being utilized in the maturation of the *PFKFB3*::*DMD* transcript.

## 3. Discussion

This case is an example of the successful use of whole genome sequencing (WGS) to end the diagnostic odyssey for two patients in a single family where other more targeted approaches failed due to the nature of the assays. Each of the approaches utilized will be discussed in terms of why they failed and how WGS overcame those limitations.

The targeted comprehensive muscular dystrophy (CMD) and neuromuscular (NM) panel was performed at Invitae (San Francisco, CA, USA) and did not return any pathogenic variants but did reveal three variants of uncertain significance (VUS): *MYL2* (c.710A>G; p.Lys237Arg), *MYL2* (c.141C>A; p.Asn47Lys), and *TMEM43* (c.403G>A; p.Glu135Lys) in the proband. The proband’s affected brother also had the CMD and NM panel showing two heterozygous benign pseudodeficiency alleles in the *GAA* gene (c.1726G>A, p.Gly576Ser and c.2065G>A, p.Glu689Lys). None of these variants were thought to be the cause of either boy’s elevated CPK levels.

The comprehensive muscular dystrophy and neuromuscular panel utilized a hybridization-based capture method that enriched 230 genes associated with muscular dystrophy or neuromuscular disorders. As with most gene panels, the intronic regions are not captured by this method. For most cases of DMD that result from frameshifts, nonsense, splice acceptor/donor, or exonic deletions or duplication mutations, this approach is suitable for identifying a pathogenic variant. However, when the causal variant is intronic, as is the case here, exon capture methods will fail to identify the variant. All the information gained from the targeted panel is also captured by the WGS data, albeit at a higher cost than the panel.

Whole exome sequencing (WES) is a technique that also utilizes exon capture methods but does so in a genome-wide manner, allowing for much greater data acquisition, but intronic regions are still omitted. While insertions can be identified using this technique, the insertions must occur within exons or very near the exons. In this case, sequencing every exon of the *DMD* gene did not identify the causal variant because the insertion of 10p15.1 is too distal from exon 45 to be captured in this assay. Furthermore, while the coding regions of the genes that were inserted in the *DMD* gene would have been captured by the test, the only conclusion that could be drawn about them is that each seems to be in a three-copy state. This is because the breakpoints of the insertion on chromosome 10 are also intronic of their respective genes, and thus, a readthrough to the X chromosome would not be expected to be observed by a WES assay. As with the targeted muscular dystrophy panel, all the information gained from WES is also captured by the WGS data, though at an elevated cost compared with WES.

Chromosomal microarray (CMA) was another genome-wide approach used to identify potential causal variants. For muscular dystrophy, these would generally be copy number losses of known dystrophy-associated genes. With a typical resolution between 5 kb and 10 kb, the 8 bp deletion in the intronic region of the *DMD* gene would never be observed. After a second review of the raw data was requested to specifically look for the copy number variant within 10p15.1 identified by WGS, the test did reveal a copy number gain of this region. However, because a CMA is incapable of determining the genomic location of identified copy number variants, the gain was not deemed pathogenic and thus was not reported. When considering the context of the patient’s phenotype and the limitations of the assay, this result is perfectly acceptable. It is not the copy number gain that is the causal variant, but because that copy number gain exists within the 44th exon of the *DMD* gene that lends pathogenicity. As with the two previous assays discussed, the information gained by performing the CMA is also captured by the WGS data but also includes location-specific information and increased resolution.

Prior to TGen’s RNA sequencing, an external clinical research laboratory performed RNA sequencing of a muscle biopsy from the proband and reported no aberrant splicing events across 133 genes associated with neuromuscular disorders. However, after the insertion was discovered at TGen, a review of the original data was requested with specific details about where to look (in intron 44) and what to expect (insertion of a portion of 10p15.1). The external laboratory responded that, after further review, aberrant splicing was observable between exons 44 and 45 of the *DMD* gene (transcript Dp427m) and that the extra genetic material was identifiable. They amended their report and provided the new version to the proband’s healthcare providers.

The RNA sequencing performed at TGen utilizing the remaining muscle biopsy further confirmed that splicing between exons 44 and 45 of the *DMD* gene was eliminated. It further added that a fusion transcript was detected, *PFKFB3*::*DMD*, with reads spanning exon 1 of *PFKFB3* to exon 45 of *DMD*, providing even more conclusive evidence that the 306 kb inverted insertion of 10p15.1 is the pathogenic variant that results in Duchenne muscular dystrophy in both boys.

This case serves as an example of the value of whole genome sequencing, and while the typical genetic tests used to diagnose DMD can work well in most cases, rarer pathogenic causes of DMD require more comprehensive approaches. Prior to WGS, the proband had four separate genetic tests performed: CMA, WES, targeted transcriptome analysis, and a comprehensive muscular dystrophy panel; all were returned with either negative results or variants of uncertain significance. With the exception of the targeted transcriptome analysis of the proband’s muscle biopsy, the results of the CMA, WES, and the dystrophy panel can all be found in the WGS data, which can reveal additional variant types unobservable by these other methods, such as the translocation identified here. It is also worth noting that karyotyping, one of the gold standard tests to identify chromosomal rearrangements, would likely have missed the inserted 10p15.1 section because its length of approximately 360 kb is well below the 5 Mb resolution of standard karyotyping [10].

At TGen, the inserted region of chromosome 10 was not observed to be deleted from the autosome in either the sons or the mother by manual analysis. In fact, the CNV caller identified that each of the probands and their mother had three copies of this region in their genomes, which was concordant with the manual review of the data. This seemed to be contradictory to the results of the CMA performed at the outside laboratory, which was returned with a result of “Negative”, as no clinically significant copy number changes were observed. However, the laboratory was able to confirm that a third copy of the 306 kb portion of 10p15.1 was observed in the CMA performed on the proband but was not reported due to a lack of genotype–phenotype correlation. As CMAs cannot identify the genomic location of extra genetic material, the original results of the CMA are understandable in the context of the genotype–phenotype correlation. Essentially, the pathogenic variant was observed, but without location information, no pathogenicity could be ascribed.

Of note, while language delay was observed in the proband’s half-brother, no other cognitive phenotypes associated with the lack of the Dp140 dystrophin isoform [1] were observed in either of the brothers. It is currently unknown if the Dp140 isoform can be generated because the 10p15.1 insertion disrupts the Dp140 regulatory elements, as the insertion falls between non-coding exons 1 and 2 of the Dp140 isoform. Though unlikely, it may also be possible that the Dp140 isoform is generated from the *PFKFB3*::*DMD* fusion mRNA (though this transcript likely undergoes nonsense-mediated decay, discussed below) or by another mechanism not requiring the first non-coding exon. However, the most likely explanation is that the transcription of the Dp140 isoform is disrupted and that this may influence the language delay observed in the proband’s half-brother. Because Dp140 is only expressed in the central nervous system and the kidneys [1], tissue was not retrieved for transcriptomic or proteomic analysis of the Dp140 isoform.

Furthermore, the RNA molecule generated from the *PFKFB3*::*DMD* translocation has 19 reads that span from exon 1 of *PFKFB3* transcripts NM_001145443.3 or NM_001323016.3 to exon 45 of the *DMD* Dp427m transcript. This mRNA has no in-frame codons downstream of exon 1 of the *PFKFB3* gene and terminates at codon 81. Three rules have been determined to account for up to 80% of reduced expression of mutant genes due to pre-mature stop codons: 1. The stop codon should be more than 54 base pairs from the final splice junction; 2. The gene has multiple exons; and 3. The stop codon is more than 200 bp downstream of the start codon [11]. Because this mRNA fulfills all three of these rules, it is predicted to undergo nonsense-mediated decay and is not expected to produce a protein that contributes to the proband’s phenotype.

In conclusion, targeted genomic approaches used to diagnose rare yet well-understood disorders can fall short of their goals when the biological cause of the disorder is rare. In this case, WGS provided the same data as the CMA, the targeted neuromuscular panel, and whole exome sequencing combined, along with the full intronic sequences that were required to locate the causal variant. Interestingly, the transcriptome splicing analysis was the one external test performed prior to WGS at TGen that should have identified the causal variant (at least at the RNA level) but failed to do so, likely due to a lack of manual interpretation of the data. However, it is worth pointing out that the ability to call the variant at the RNA level was inferred from the WGS data, highlighting the importance of a comprehensive genomic workup in rare disorders that lack a genetic diagnosis. For DMD patients, it is imperative that the genetic cause is identified to qualify for a gene therapy clinical trial or FDA-approved therapeutic to ensure that both the muscular dystrophy observed is Duchenne and the specific mutation is amenable to the mechanism of action of the therapeutic.

## 4. Materials and Methods

### 4.1. Whole Genome Sequencing (WGS)

Whole genome sequencing was performed at the Translational Genomics Research Institute, part of City of Hope, in Phoenix, Arizona. Blood was drawn from both boys, their mother, and each of their fathers at the clinic, then sent to TGen Clinical Lab for DNA extraction. Extracted DNA was transferred to TGen’s Collaborative Sequencing Center for library preparation with a PCR-free workflow using the Watchmaker DNA Library Prep Kit with Fragmentation (Watchmaker Genomics, Denver, CO, USA) using 100 ng DNA input. Libraries were sequenced on the NovaSeq X Platform (Illumina, San Diego, CA, USA), using the 2 × 150 bp chemistry, to a target coverage of 30X.

### 4.2. WGS Analytical Pipeline

FASTQs were generated using BCL Convert (Illumina, San Diego, CA, USA) and aligned to GRCh37/hg19 [12] using bwa-mem2 release 2.2.1 [13] to generate the binary alignment maps (BAMs), which were processed with SAMtools 1.10 [14] through sort, fixmate, and markdup. Genomic variant call format files (GVCFs) were generated from the BAMs using HaplotypeCaller, combined using CombineGVCFs, and then jointly called using GenotypeGVCFs, all packages from GATK 4.1.8.0 [15]. CNVkit v0.9.10 [16] and Manta 1.6 [17] were used to produce copy number and structural variant call format files (VCFs), respectively. The median coverage of the five genomes was 27X. HaplotypeCaller VCFs were analyzed for variants using VarSeq (Golden Helix Inc., Bozeman, MT, USA), and the Emedgene AI platform (Illumina, San Diego, CA, USA) was utilized using the HaplotypeCaller and CNVkit VCFs. VCFs from Manta were not utilized in either platform.

### 4.3. WGS Manual Analysis

Because of the strong suspicion of DMD in both boys, the *DMD* gene was manually reviewed. VCFs from Manta, an algorithm used to identify structural variants and indels, along with the alignment BAMs from all five sequenced individuals, were uploaded into the Integrated Genomics Viewer (IGV) version 2.14.1 [18]. The *DMD* gene was manually searched for any potentially causal variants.

### 4.4. Confirmation of Translocation

Aliquots of both boys’ and the mother’s blood were sent from TGen Clinical Lab to Greenwood Genetic Center for confirmation of the inverted insertion of approximately 306 kb of 10p15.1 into intron 44 of the *DMD* gene via distal and proximal breakpoint sequencing.

### 4.5. RNA Sequencing (RNA-Seq)

RNA-seq was performed at TGen using a portion of the proband’s muscle biopsy that remained after the original splicing analyses were performed. RNA was extracted using the Maxwell AS1340 RSC Simply RNA Tissue kit (Promega, Fitchburg, WI, USA) and the Quick-RNA Miniprep Plus kit (Zymo Research, Tustin, CA, USA). The RNA extractions were combined, cleaned with the Nucleospin RNA Clean-up kit (Takara Bio, San Jose, CA, USA), and subjected to quality control using the Qubit BR RNA assay (Thermo Fisher Scientific, Waltham, MA, USA) and the TapeStation RNA ScreenTape assay (Agilent, Santa Clara, CA, USA). The purified RNA was submitted to TGen’s Collaborative Sequencing Center for library preparation using the Kapa RNA HyperPrep with RiboErase (Roche, Basel, Switzerland) kit, and the library was quality controlled using the Qubit High Sensitivity dsDNA assay (Thermo Fisher Scientific, Waltham, MA, USA) and the High Sensitivity D1000 ScreenTape assay (Agilent, Santa Clara, CA, USA). The library was sequenced on the NovaSeq X Plus platform (Illumina, San Diego, CA, USA), which resulted in over 191 million paired end reads.

### 4.6. RNA-Seq Analysis

FASTQs were generated from the raw RNA sequencing data using Bcl2Fastq v2.20.0.422 (Illumina), which were aligned to the human genome (GRCh38/hg38) [19] using the STAR aligner v.2.7.8a [20] to generate the BAMs. BAMs were sorted, and mate coordinates were fixed using SAMtools v1.17 [14]. PCR duplicates were identified using Picard MarkDuplicates v4.4.0.0 [21], and then the BAMs were compressed to CRAMs using SAMtools v1.17 [14]. STAR-Fusion 1.11.0 [22] was utilized for fusion calling. Sashimi plots were generated using IGV v.2.14.1 [18] and included RNA-seq data from a commercially sourced, normal human skeletal muscle specimen from BioIVT (Hicksville, NY, USA; Cat # 99623-1210333F) previously sequenced (at TGen) utilizing the same RNA library preparation kit as the proband’s specimen.

## Data Availability

The data presented in this study are available on request from the corresponding author due to privacy reasons.

## References

[1] Duan D., Goemans N., Takeda S., Mercuri E., Aartsma-Rus A. (2021). Duchenne muscular dystrophy. Nat. Rev. Dis. Primers.

[2] Gao Q.Q., McNally E.M. (2015). The Dystrophin Complex: Structure, function and implications for therapy. Compr. Physiol..

[3] Koenig M., Kunkel L.M. (1990). Detailed analysis of the repeat domain of dystrophin reveals four potential hinge segments that may confer flexibility. J. Biol. Chem..

[4] Houang E.M., Sham Y.Y., Bates F.S., Metzger J.M. (2018). Muscle membrane integrity in Duchenne muscular dystrophy: Recent advances in copolymer-based muscle membrane stabilizers. Skelet. Muscle.

[5] Emery A.E.H. (2002). The muscular dystrophies. Lancet.

[6] Mareedu S., Million E.D., Duan D., Babu G.J. (2021). Abnormal Calcium Handling in Duchenne Muscular Dystrophy: Mechanisms and Potential Therapies. Front. Physiol..

[7] Kim E.Y., Lee J.W., Suh M.R., Choi W.A., Kang S.W., Oh H.J. (2017). Correlation of Serum Creatine Kinase Level with Pulmonary Function in Duchenne Muscular Dystrophy. Ann. Rehabil. Med..

[8] Nabuurs C.I., Choe C.U., Veltien A., Kan H.E., van Loon L.J.C., Rodenburg R.J.T., Matschke J., Wieringa B., Kemp G.J., Isbrandt D. (2013). Disturbed energy metabolism and muscular dystrophy caused by pure creatine deficiency are reversible by creatine intake. J. Physiol..

[9] Aartsma-Rus A., Ginjaar I.B., Bushby K. (2016). The importance of genetic diagnosis for Duchenne muscular dystrophy. J. Med. Genet..

[10] di Gregorio E., Savin E., Biamino E., Belligni E.F., Naretto V.G., D’alessandro G., Gai G., Fiocchi F., Calcia A., Mancini C. (2014). Large cryptic genomic rearrangements with apparently normal karyotypes detected by array-CGH. Mol. Cytogenet..

[11] Hu Z., Yau C., Ahmed A.A. (2017). A pan-cancer genome-wide analysis reveals tumour dependencies by induction of nonsense-mediated decay. Nat. Commun..

[12] Church D.M., Schneider V.A., Graves T., Auger K., Cunningham F., Bouk N., Chen H.C., Agarwala R., McLaren W.M., Ritchie G.R.S. (2011). Modernizing reference genome assemblies. PLoS Biol..

[13] Md V., Misra S., Li H., Aluru S. Efficient architecture-aware acceleration of BWA-MEM for multicore systems. Proceedings of the 2019 IEEE 33rd International Parallel and Distributed Processing Symposium, IPDPS.

[14] Danecek P., Bonfield J.K., Liddle J., Marshall J., Ohan V., Pollard M.O., Whitwham A., Keane T., McCarthy S.A., Davies R.M. (2021). Twelve years of SAMtools and BCFtools. GigaScience.

[15] Poplin R., Ruano-Rubio V., DePristo M.A., Fennell T.J., Carneiro M.O., van der Auwera G.A., Kling D.E., Gauthier L.D., Levy-Moonshine A., Roazen D. (2018). Scaling accurate genetic variant discovery to tens of thousands of samples. bioRxiv.

[16] Talevich E., Shain A.H., Botton T., Bastian B.C. (2016). CNVkit: Genome-Wide Copy Number Detection and Visualization from Targeted DNA Sequencing. PLoS Comput. Biol..

[17] Chen X., Schulz-Trieglaff O., Shaw R., Barnes B., Schlesinger F., Källberg M., Cox A.J., Kruglyak S., Saunders C.T. (2016). Manta: Rapid detection of structural variants and indels for germline and cancer sequencing applications. Bioinformatics.

[18] Robinson J.T., Thorvaldsdóttir H., Wenger A.M., Zehir A., Mesirov J.P. (2017). Variant review with the integrative genomics viewer. Cancer Res..

[19] Schneider V.A., Graves-Lindsay T., Howe K., Bouk N., Chen H.-C., Kitts P.A., Murphy T.D., Pruitt K.D., Thibaud-Nissen F., Albracht D. (2016). Evaluation of GRCh38 and de novo haploid genome assemblies demonstrates the enduring quality of the reference assembly. bioRxiv.

[20] Dobin A., Davis C.A., Schlesinger F., Drenkow J., Zaleski C., Jha S., Batut P., Chaisson M., Gingeras T.R. (2013). STAR: Ultrafast universal RNA-seq aligner. Bioinformatics.

[21] Picard. https://broadinstitute.github.io/picard/.

[22] Haas B.J., Dobin A., Li B., Stransky N., Pochet N., Regev A. (2019). Accuracy assessment of fusion transcript detection via read-mapping and de novo fusion transcript assembly-based methods. Genome Biol..

